# Success and patient satisfaction of immediately loaded zirconia implants with fixed restorations one year after loading

**DOI:** 10.1186/s12903-022-02231-0

**Published:** 2022-05-23

**Authors:** Rico Rutkowski, Ralf Smeets, Leon Neuhöffer, Carolin Stolzer, Kilian Strick, Martin Gosau, Susanne Sehner, Karl Ulrich Volz, Anders Henningsen

**Affiliations:** 1 Department of Oral and Maxillofacial Surgery, University Medical Center Hamburg-Eppendorf, Martinistrasse 52, 20246 Hamburg, Germany; 2 Division Regenerative Orofacial Medicine, Department of Oral and Maxillofacial Surgery, University Medical Center Hamburg-Eppendorf, Martinistrasse 52, 20246 Hamburg, Germany; 3 Department of Medical Biometry and Epidemiology, University Medical Center Hamburg-Eppendorf, Hamburg, Germany; 4 Swiss Biohealth Clinic, Kreuzlingen, Switzerland

**Keywords:** Zirconia, Dental implants, Immediate placement, Immediate loading, Ceramic

## Abstract

**Background:**

There is limited evidence for the use of zirconium dioxide implants in immediate implant placement as well as for related immediate loading protocols. The aim of this retrospective study was to investigate the survival rate, success and patient satisfaction of immediately placed zirconia implants compared to delayed placed implants.

**Methods:**

The study included 58 partially edentulous patients who were treated between 2013 and 2015 with immediate and delayed transgingival healing zirconium dioxide implants (SDS, Kreuzlingen/ Switzerland). In addition to survival and success rate, marginal bone loss was assessed using radiographs and soft tissue was evaluated using Pink Esthetic Score. Oral health-related quality of life was investigated prospectively using a modified OHIP questionnaire.

**Results:**

The cumulative survival rate of all implants included was 92% with 88% classified as full success. No significant difference was found between the bone levels of immediately and delayed placed and immediately and delayed loaded implants. The mean Pink Esthetic Score after final prosthetic rehabilitation was 12.2/14 points indicating excellent esthetic clinical results. Analysis of the OHIP questionnaire showed a mean value of 0.54/100 points reflecting a high patient satisfaction.

**Conclusions:**

Immediate and delayed placed as well as loaded zirconium dioxide implants showed excellent results regarding implant success and survival in this study. Zirconium dioxide implants may ensure excellent esthetic results and high patient satisfaction.

**Supplementary Information:**

The online version contains supplementary material available at 10.1186/s12903-022-02231-0.

## Introduction

Since the discovery of osseointegration by Per-Ingvar Brånemark in the 1970s, a stress-free healing period of 3–6 months was assumed as one of the most important requirements for a predictable treatment success in dental implantology [1]. The first immediate loading protocol was successfully tested in the 1980s with implant-retained overdentures in fully edentulous patients, which was later adapted to fixed implant-supported prosthesis [2]. The introduction of optimized microrough surfaces in dental implantology opened up the possibility to restore single tooth spaces using a one-stage procedure and to increase attractiveness of dental implant therapy [3]. Several research groups reported implant survival rates for such a protocol over 90% after 7 and 10 years and showed that immediate implant placement may be a reliable treatment option [4–6].

However, tooth extraction is followed by bone remodeling that takes place in the extraction socket and may be a risk factor for immediate implant placement [7].

A more advanced approach is the immediate restoration of implants. The results of in-vivo studies showed that immediately loaded implants can achieve an increased or at least equivalent Bone-to-Implant Contact (BIC) compared to conventionally loaded implants [8]. The same study showed that bone density of immediately loaded implants may even be higher compared to conventionally loaded implants [8]. Nevertheless, immediate loading appears to be an individual option in carefully chosen cases in order to avoid early implant failures [9]. Taking certain requirements into account, immediate loading of dental implants has been described as a reliable concept in the consensus statement of Gallucci et al. [10]. Benefits for the patient may be a reduced treatment duration, reduced number of surgical interventions and a shortened time between extraction and prosthetic rehabilitation which may result in higher patient satisfaction and acceptance [11]. In comparison with other implant placement protocols, no differences in terms of success rates could be found, even considering implant sites with local pathologies or periodontal disease [12]. Studies showed that bone loss and esthetic outcome may be similar to conventional implant loading protocols in titanium implants [4].

Due to its outstanding esthetic properties and excellent biocompatibility, dental implants made of zirconium dioxide have recently come more into focus. In 1993, the first successful osseointegration of zirconium dioxide implants in mammals was shown by Akagawa et al. [13]. Further studies showed that implants made of zirconium dioxide may lead to clinical results that are comparable to the results of conventional titanium implants [14]. However, the number of studies, especially for immediate loading and immediate placement of zirconia implants, is still low. Since the first decade of this century, various studies have reported almost the same success rates for ceramic implants made of zirconium dioxide compared to titanium implants [15]. In vivo studies showed that peri-implant bone surrounding zirconium implants may have higher density compared to bone around titanium implants, the bone loss may be lower and the bone implant contact may be similar [14, 16]. Additionally, implants made of zirconium dioxide could be superior compared to titanium implants in terms of bacterial adhesion and may lead to lower levels of inflammation mediators in peri-implant tissues resulting in an improved esthetic outcome [17–20]. Due to the limited numbers of patients and implants, studies on ceramic implants are frequently lacking validity in terms of survival and success rates [21]. Currently, no studies exist on immediately placed and immediately loaded ceramic implants and investigations on this topic would be of particular scientific interest [22].

Therefore, the aim of this study was to investigate the survival and success rates, the esthetic outcome and patient satisfaction of immediately and delayed placed zirconium dioxide implants after immediate or conventional loading. The hypothesis was that there are no statistically significant differences regarding survival and success with respect to time of placement and loading.

## Methods

In this study, data of 58 partially edentulous patients who received a total of 163 immediately or conventionally placed one-piece and two-piece zirconium dioxide implants in different anatomical regions of the maxilla and mandible, that were immediately or conventionally loaded between June 2013 and June 2015 were analyzed. Implants were placed in three dental practices by one surgeon.

This study was performed in accordance with the Declaration of Helsinki (64th WMA General Assembly, Brazil, October 2013). All patients were at least 18 years of age and agreed to the treatment with informed consent. The research project was approved by the Ethics Committee of the Hamburg Medical Association (Reg. Nr. PV5074).

The inclusion criteria were adapted to the requirements of the fifth ITI consensus conference [10]: (1) implants placed in the upper and lower jaw between June 2013 and June 2015; (2) primary stability with torque values ≥ 20 Ncm to 45 Ncm; (3) lack of local contraindications such as parafunctions and extensive bone defects; (4) the clinical benefits outweigh the risks.

The exclusion criteria were defined as follows: (1) Compromised general health, which may have an effect on the healing and osseointegration of dental implants, according to recommendations from the second ITI consensus conference [23]; (2) parafunctions (bruxism, clenching, etc.); (3) heavy smokers (> 10 cigarettes per day); (4) malignant disease; (5) chemotherapy, bisphosphonate therapy, irradiation therapy; (6) immunocompromising diseases, *e.g.* HIV; (7) poor oral hygiene (PSI ≥ 3).

The requirements for immediate implant placements were according to the third ITI Consensus Conference [11]: (1) minimal-invasive tooth extraction, in particular with retention of the vestibular bone lamella; (2) removal of all granulation tissue; (3) sufficient primary stability (30–40 Ncm); (4) three-dimensionally correct position considering the later restoration by means of the provisional tool based on the previous tooth position, (5) no local contraindications like parafunctions or extensive bone defects and (6) the outweighing of clinical benefits in comparison to associated risks.

### Surgical procedure

The necessary implant parameters and the need for augmentative procedures were determined on the basis of a thorough preoperative examination and diagnostic procedures using orthopantomograms or digital volume tomography and intraoral photography. All patients were treated under local anesthesia. Only SDS implants (Swiss Dental Solutions AG, Kreuzlingen, Switzerland) were used. These implants are made of alumina toughened zirconia (ATZ) or tetragonal zirconia polycrystal (TZP). All implants possess a TZP Zircapore® surface (Metoxit AG, Thayngen, Switzerland) with a roughness of averagely 2 µm.

The tooth removal prior to immediate implant placement was carried out as atraumatically as possible. Special attention was paid to the preservation of the vestibular bone lamella. After removal of the tooth, the sockets were carefully curetted to ensure that no infectious tissue remained inside. In cases of conventionally placed implants, the gingiva was punched in the diameter of the planned implant diameter. In cases of perioperative augmentation in the maxilla, a mucoperiosteal flap was elevated after crestal incision and an augmentation of the maxillary sinus was performed by placing a vestibular bone window between the canine fossa and zygomaticoalveolar crest using Piezosurgery® touch (mectron Germany Vertriebs GmbH, Cologne, Germany) and augmenting a mixture of chronOS™ (pure beta tricalcium phosphate, Depuy Synthes, Zuchwil, Switzerland) and autologous PRGF (Plasma Rich in Growth Factors).

### Immediate loading protocol

All immediately loaded implants were supplied with a chairside provisional restoration within 24 h using either Luxatemp® (DMG, DMG Chemisch-Pharmazeutische Fabrik GmbH, Hamburg, Germany) or Protemp™ (3 M ESPE, 3 M Deutschland GmbH, Neuss, Germany). To avoide lateral shearing forces, static and dynamic occlusal contacts were carefully removed and group guidance was created in the partial edentulous jaws. All patients were instructed to take a soft food diet in the area of the long-term provisional restoration for three months and avoiding chewing or biting in cases of anterior implant placement.

### Data collection

The average radiographic peri-implant radiological bone level was measured by means of using pre- and postoperative X-rays radiographs. Additionally, the soft tissue ratios of 30 immediately placed implants were determined using the Pink Esthetic Score (PES). Health-related quality of life of 41 patients was assessed after prosthetic supply provision using a modified OHIP questionnaire.

### Radiographic measurements

Measurements of the bone height were performed using orthopantomograms. In two of the treatment centers integrated in this study, only digital radiographs were performed and analyzed. The analog images of the third treatment center were digitized before analysis using an Epson Expression 1680 (EPSON Deutschland GmbH, Meerbusch, Germany). All x-ray images were evaluated using DBSWIN software, release 5.5.0 (Dürr Dental AG, Bietigheim-Bissingen, Germany). All images were measured by two independent researchers not part of the study team. Images were assessed in a darkened room on a diagnostic monitor (Philips 220WS8, Philips, Amsterdam, Netherlands). Recalls as well as radiographic checks were performed on a semi-annual basis and were recorded up to 1.5 years after implant placement. Orthopantomograms were taken directly after implant placement and during the follow-up visits. The included follow up data extend up to 1.5 years after implant placement.

Images were calibrated and the distance between the bone level from the reference point to the implant was measured mesially and distally and then arithmetically averaged. According to Buser et al., the crestal bone height was defined as the most coronal bone-to-implant contact [24]. Postoperative radiographs served as baseline. The bone level at each follow-up radiograph was subtracted from the baseline to obtain changes in bone level.

### Success criteria

The implant success was defined according to the criteria defined by the Health Scale for Dental Implants (Table 1), presented at the Pisa Consensus Conference, Italy 2007 [25].

**Table 1:** Criteria for implant success

Implant quality scale group	Clinical conditions
Success (optimum health)	No pain or tenderness upon function 0 mobility < 2 mm radiographic bone loss from initial surgery No exudates history
Satisfactory survival	No pain on function 0 mobility 2–4 mm radiographic bone loss No exudates history
Compromised survival	May have sensitivity on function 0 mobility Radiographic bone loss > 4 mm (less than ½ of implant body) Probing depth > 7 mm May have exudates history
Failure (clinical or absolute failure)	Any of following: Pain on function Mobility Radiographic bone loss > ½ length of implant Uncontrolled exudate No longer in mouth

### Pink Esthetic Score

In one of the three practices, photographical documentation accompanying all prosthetic procedures was carried out using a digital reflex camera with macro-lens and ring-flash-system (Canon Eos 400D, Macro-Ringlite, Canon Inc., Tokyo, Japan). All photos were taken immediately after prosthetic supply, mirrored and transferred into Irfanview software, release 4.38 (Irfan Skiljan, Vienna, Austria), by an independent person who was not involved in the surgical procedures. Assessment of the Pink Esthetic Score included 30 immediate implants received by 13 patients. The pictures were printed in color on a Develop ineo + 554 color printer (Konica Minolta Business Solutions Germany GmbH, Langenhagen, Germany) and were evaluated by 6 independent dentists not part of the study team to assess esthetics after prosthetic restoration compared to the situation prior to implant placement (Additional file 1: Supplementary file 1). Evaluations were repeated after 4 weeks. Seven variables such as soft tissue color and texture or recession of the alveolar process were assessed by ratings ranging from 0 to 2 points. The intra- and interobserver agreement was evaluated using Cronbach’s Alpha.

### Oral health-related quality of life

The questionnaire study was conducted using a modified OHIP Edent (Oral Health Impact Profile) questionnaire (Additional file 2: Supplementary file 2). During February and March 2016, the modified OHIP Edent questionnaire with patient information and consent form was sent to 41 patients on average 21 months after their operation (range 10–32 months). The results were digitized in Excel (Microsoft Office version 2013, Microsoft Corporation, Redmond, USA) and assigned to the relevant data record.

### Statistical analysis

The collected data was transferred into an Excel chart (Microsoft Office version 2013, Microsoft Corporation, Redmond, USA). After descriptive analysis of the data, Stata was used for statistical analysis (StataCorp LP, College Station, Texas, USA). A likelihood ratio test in which mixed models with measurement repeatability were checked for certain restrictions was used to determine significant differences. Cronbach’s Alpha was used to verify the intra- and interobserver agreement of the Pink Esthetic Score.

## Results

### Descriptive data

The data of 58 patients with 163 implants was acquired, 7 patients with 13 implants dropped out due to lack of follow up data. Most implants (44.7%) were placed in the posterior upper jaw, the fewest (3.3%) to replace lower incisors (Table 2).

**Table 2:** Distribution of implants

Implant region according to Fédération Dentaire Internationale (FDI)	1	2	3	4	5	6	7	8
	Anterior			Posterior				
Maxilla total	14 (9.3%)	8 (5.3%)	7 (4.7%)	24 (16%)	17 (11.3%)	18 (12%)	8 (5.3%)	-
Mandibula total	1 (0.7%)	1 (0.7%)	3 (2%)	7 (4.7%)	13 (8.7%)	21 (14%)	8 (5.3%)	-
Immediate loading	15 (10%)	9 (9%)	9 (9%)	27 (18%)	27 (18%)	16 (10.7%)	10 (6.7%)	-
Delayed loading	0	0	1 (0.7%)	4 (2.7%	3 (2%)	23 (15.3%)	6 (4%)	-
Immediate implant placement	13 (8.7%)	8 (5.3%)	9 (9%)	28 (18.7%)	23 (15.3%)	12 (8%)	8 (5.3%)	-
Delayed implant placement	1 (0.7%)	1 (0.7%)	1 (0.7%)	3 (2%)	7 (4.7%)	17 (11.3%)	8 (5.3%)	-

**Fig. 1:**
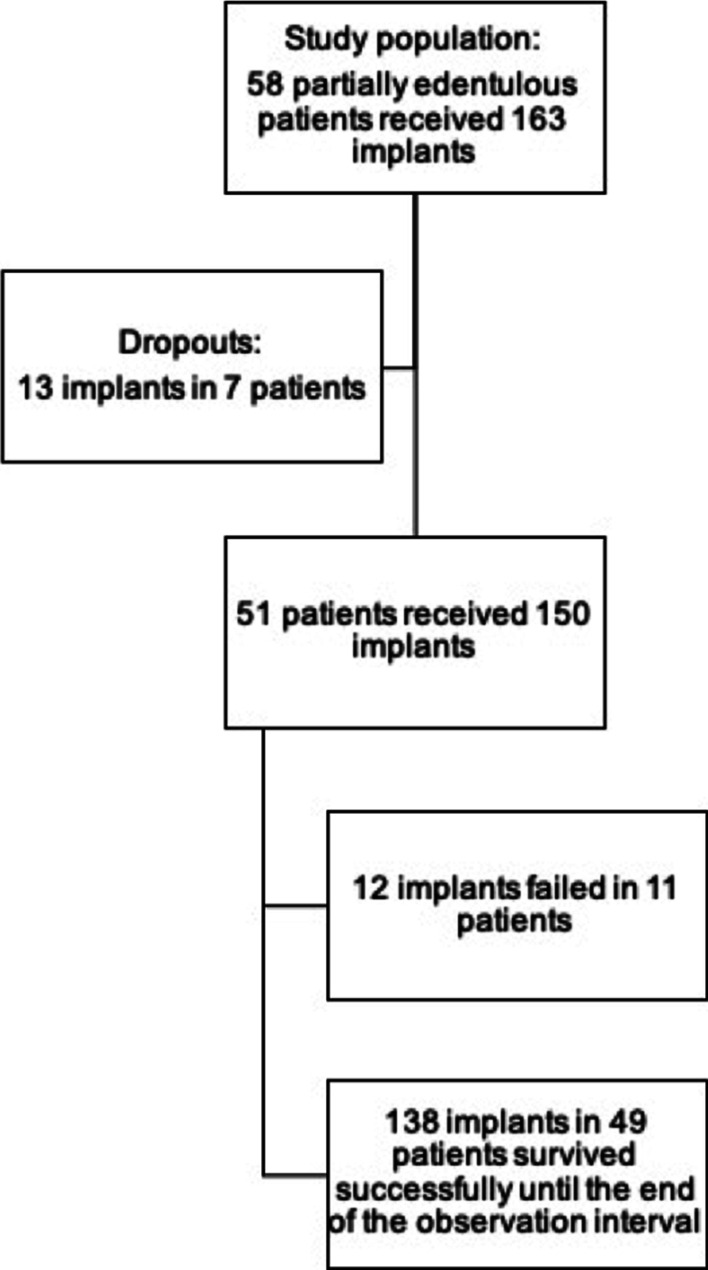
Flowchart representing study population, dropouts, failures and successful surviving implants

A total of 150 implant placements, including 96 maxillary and 54 mandibular implants, were evaluated. One-hundred-twenty-nine implants (86%) were one-piece zirconia implants. The patient collective consisted of 51 patients with a mean age of 57 years (Fig. 1). Thirty-four patients received 82 immediate implants and 9 implants were conventionally placed in 3 patients. Fourteen patients received 29 immediate as well as 30 conventionally placed implants. In total, 113 implants (75.3%) in 44 patients were loaded immediately and 37 implants (24.7%) in 30 patients were conventionally loaded after implant placement (Table 3). In 16 patients, implants with both types of loading were used. The time difference from implant placement to final prosthetic restoration was 7 months on average for immediately placed implants and 8.7 months on average for conventionally placed implants.

**Table 3:** Success rates of implants. Please note that 13 patients received implants with both types of placement and both types of loading were used in 16 patients

		Success per implant (last recall)				
		Implant Quality Scale Group I (Success)	Implant Quality Scale Group II (Satisfactory survival)	Implant Quality Scale Group III (Compromised survival)	Implant Quality Scale Group IV (Failure)	Total
Time of implant placement	Immediate	98 (65.3%) in 35 patients	4 (2.7%) in 3 patients	0	11 (7.3%) in 10 patients	113 (75.3%)
	Delayed	34 (22.7%) in 13 patients	1 (0.7%) in 1 patient	1 (0.7%) in 1 patient	1 (0.7%) in 1 patient	37 (24.7%)
	Total	132 (88%)	5 (3.3%)	1 (0.7%)	12 (8%)	150 (100%)
Provisional loading	Immediately	103 (68.7%) in 35 patients	2 (1.3%) in 2 patients	1 (0.7%) in 1 patient	7 (4.7%) i n 6 patients	113 (75.3%)
	Delayed	29 (19.3%) in 23 patients	3 (2%) in 2 patients	0	5 (3.3%) in 5 patients	37 (24.7%)
	Total	132 (88%)	5 (3.3%)	1 (0.7%)	12 (8%)	150 (100%)

In 3 implant sites (one patient), sinus augmentation was performed prior to implant placement as separate intervention using chronOS™ (Depuy Synthes Companies, Zuchwil, Switzerland). Twelve implants were placed accompanied by grafting procedures. In 3 of these cases (2 patients), simultaneous sinus augmentation using CEROS® (Thommen Medical AG, Grenchen, Switzerland) and in 9 cases (2 patients), sinus augmentation using chronOS™ (Depuy Synthes Companies, Zuchwil, Switzerland) was performed. Six of those implants were immediately and 6 implants were conventionally loaded. Only one implant accompanied by these grafting procedures was lost (immediately loaded).

The recall took place in three periods: in the first half year after implant placement 40 patients with 107 implants completed 139 recalls, between a half and one year 19 patients with 60 implants accomplished 72 recalls and one to one and half years after implant placement 14 patients with 45 implants showed up to 65 recalls.

### Success rates

Among 150 implants, 132 implants were successful, giving a success rate of 88% and failure rate of 8% (n = 12 implants, Table 3).

### Prosthetic restoration

Fifty-three implants (38.4%) received single crowns and 20 implants (14.5%) were suppled using implant-supported bridges. Due to the manufacturer’s specification that prosthetics on neighboring implants have to be connected to each other, 65 implants (47.1%) were supplied using splinted crowns. Twenty-four patients received only single-crown restorations, 1 patient only an implant-supported bridge and 11 patients only splinted crowns. Mixed solutions were used in 23 patients. Two patients would have been supplied using single crowns only, but implants failed.

### Failures

A total of 12 implants (8%) failed in 11 patients (21.6%) by the end of the follow up period. Eleven immediately placed implants (7.3%) and one implant following delayed placement (0.7%) failed. Seven of the failures were loaded immediately and 5 of the failed implants were delayed loaded. Two of the failed implants (16.7%) were two-piece zirconia implants and 10 of the failures had a one-piece design. The average time until implant loss with respect to all implants lost was 125 days.

### Bone level

**Fig. 2:**
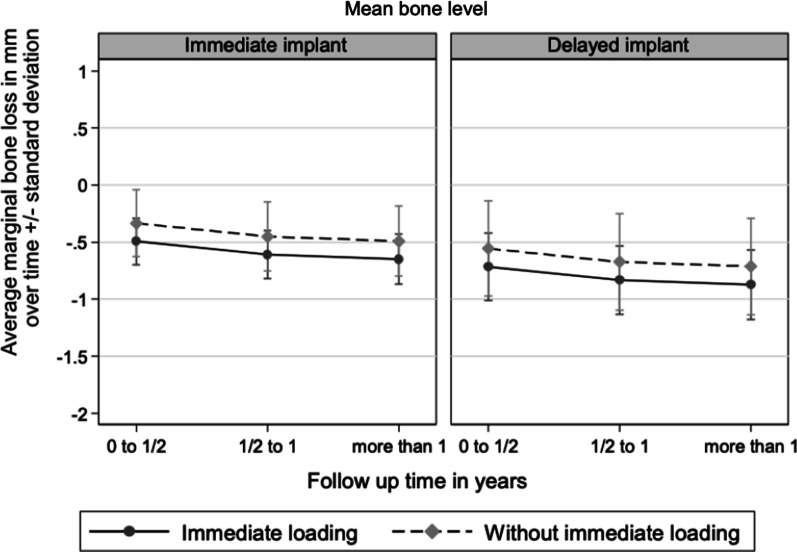
Time-dependent mean radiographic implant bone level decrease with respect to placement time

The peri-implant bone level decreased by 0.58 ± 0.77 mm in immediately placed implants and by 0.73 ± 1.16 mm in conventionally placed implants at the time of the last recall (Fig. 2).

### Pink Esthetic Score

A total of 30 implants in 13 patients were evaluated by six independent dentists with different specializations. Data of 45 implants could not be included due to lacking image quality, faulty camera settings or missing images, limiting the data significance. The mean value of the Pink Esthetic Score is 12.14 points at the time of the first measurement and 12.3 points at the time of the second measurement. The intra- and interobserver reliability of the Pink Esthetic Score has been assessed via Cronbach`s Alpha. The variables soft tissue contour, color and texture, distal papilla as well as alveolar process deficiency showed no internal consistency with respect to intra-observer agreement with a value < 0.7 in contrast to the variables mesial papilla (0.808) and marginal gingiva height (0.742). Only the variable soft tissue texture (0.563) showed a lack of internal consistency with respect to inter-observer agreement.

### Oral health-related quality of life

Ten of the patients whose data was evaluated could not be reached or refused to receive the questionnaire in advance. It was sent or given to 41 patients who received either immediately placed or immediately and conventionally placed implants. Fourteen patients did not respond at all and 5 patients refused to participate after receiving the questionnaire giving a return rate of n = 22 (53.7%). Overall, an average of 0.54 points was obtained.

### Statistical results

**Fig. 3:**
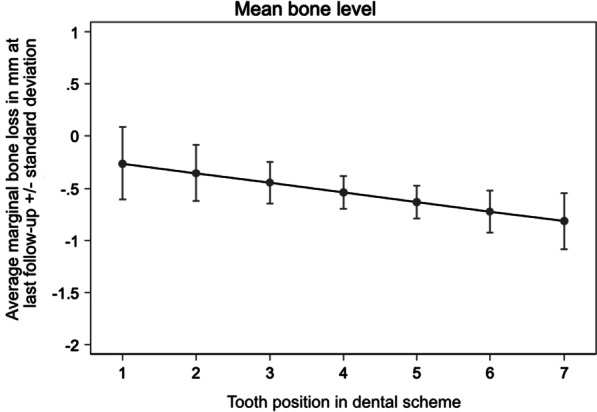
Characterization of the differences in time dependent marginal bone loss since baseline with respect to implant region

No correlation was found between marginal bone level and placement time (*p* = 0.225), type of loading (*p* = 0.192), implant width (*p* = 0.419), implant design (*p* = 0.322) and implant length (11 mm: *p* = 0.217, 14 mm: *p* = 0.862). A statistically significant effect regarding the time-dependent behavior of the marginal bone level was determined for the time periods of 0.5–1 year (*p* = 0.023) and > 1 year (*p* = 0.011) compared to the time period of up to 0.5 years after placement. Additionally, there was a statistically significant effect showing a decrease of 0.09 mm in marginal bone the further distally the implant position was with respect to dental arch (Fig. 3). No significant correlation was found between implant success and time point of placement (*p* = 0.17) as well as loading (*p* = 0.705).

## Discussion

The aim of this study was to investigate the survival and success rates, the esthetic outcome and patient satisfaction of immediately and delayed placed zirconium dioxide implants after immediate or conventional loading. The hypothesis was that there are no statistically significant differences regarding survival and success with respect to time of placement and loading. After a maximum follow up period of approximately 26 months, a survival rate of 92% and a success rate of 88% (Implant Quality Scale Group I) was determined. No significant differences were found in marginal bone level loss with respect to implant placement time, provisional loading type, implant design and implant width. There was no statistically significant effect on the implant success for the time point of placement and the type of loading.

In this study, the difference of the marginal bone level loss was 0.6 mm for immediately placed implants and 0.7 mm for implants placed in completely healed bone at the last recall which was not statistically significant. Apart from this, the average bone loss was low and is comparable to the results of other studies on immediately loaded ceramic and titanium implants [26, 27]. A statistically significant effect on the marginal bone loss was obtained for the recall periods of 6 months up to 1 year after implant placement (*p* = 0.023) and beyond 1 year (*p* = 0.011) compared to the initial 6 months after implant placement. Bone loss was more pronounced in the first half of the year than in the following periods which is also comparable to the results of other publications [28, 29]. Borgonovo et al. found that single-piece ceramic implants loaded immediately after placement achieved survival and success rates of 100% after at least 12 months. In this study, as in the present study, marginal bone loss was more pronounced in the first 6 months than in the following period. Borgonovo et al. [29] reported an average bone loss in immediate loaded implants of 1.375 ± 0.388 mm from baseline to 6 months and 0.368 ± 0.387 mm in the 1–2-year period. In contrast to the results of Borgonovo et al., the implant site in the present study was a statistically significant factor for peri-implant radiological bone loss (*p* = 0.04). Although majority of studies describe a lack of evidence for implant localization as a risk factor for crestal bone loss, some studies indicate increased risks for peri-implant inflammation and marginal bone loss in posteriorly placed implants [30, 31]. One reason could be the more limited hygienizing capability in the rear area especially when chairside provisional immediate restorations are used and the restorations on neighboring implants have to be connected to each other. However, since there is a lack of studies investigating the effects of immediately placed and / or loaded implants in different jaw locations, further research by means of prospective studies should be conducted.

Although 75.3% of all implants were loaded immediately, only 7/12 immediately loaded implants (58%) failed giving a survival rate of 93.5% after 19.5 months. The survival rate for implants undergoing conventional loading was 86.5% (5 failures) after 25.7 months. Majority of implants were placed in the maxillary posterior region but mostly in premolar positions. Less than 50% of all implants that were placed in molar position, where primary stability may be difficult to achieve, were loaded immediately. In contrast, 88.5% of all implants in premolar positions were loaded immediately. However, a lack of primary stability may have contributed to the failure rate of 8% because it cannot be ruled out that the removed conventionally loaded implants were probably not immediately loaded due to insufficient primary stability. Due to the implant design of one-piece implants that were used in 86% of all cases in this study, loads cannot always be avoided which may result in micro-motions and an increased failure rate. In a recent consensus conference, different timings of implant placement and loading presented with high implant survival rates and another review showed that different loading protocols did not seem to influence esthetic outcomes in short- and medium-term follow-ups [32, 33]. However, it cannot be excluded that immediate loading in posterior areas, where masticatory forces are considerably higher, may contribute to lower implant success rates.

During the healing period, micro-motions of over 150 μm have to be avoided [34]. The combination of low primary stability and premature loading due to micro-motions of the intraorally exposed one-piece zirconia implants may be an explanation for these failures. Due to lacking data on the precise values of insertion torques, the surgeon’s decision whether to load implants immediately or not with respect to primary stability could have biased the delayed loading group towards greater failure.

In a study of Lambrich et al. all of the lost ceramic implants placed at maxillary implant sites showed a low primary stability (< 35 Ncm insertion torque) using provisional prosthetics that could not fully prevent immediate loading [35]. If the primary stability is too low, the probability of implant survival may be reduced. Many of the existing publications on zirconia implants are short-term studies with different success criteria and treatment protocols, in particular regarding the time point of placement and loading [28, 29, 35–37]. This makes it difficult to classify the results of the present study into the scientific context.

Brüll et al. determined a cumulative survival rate of 96.5% after 18.4 months in 74 patients undergoing a conventional loading protocol without immediate implant placement in 121 zirconia implants [36]. Spies et al. published a prospective cohort study with 27 alumina-toughened zirconia (ATZ) implants in 27 patients undergoing immediate loading. After one year, three implants had to be removed giving a survival rate of 88.9% and a mean marginal bone level loss of 0.77 mm was assessed [37]. In an earlier study, Kohal et al. examined 20 single-tooth zirconia implants with immediate provisional supply in 20 patients and investigated the peri-implant bone loss [26]. They found a survival rate of 90% after one year as well as an average peri-implant bone loss of 0.88 mm. No immediate implants were placed. Both studies have a very small number of cases, which may lead to non-representative results. However, the marginal bone loss and the survival rates were comparable to the results of the present study.

Grassi et al. reported a cumulative survival rate of 96.8% after 4.3–6 years in 32 immediately loaded one-piece zirconia implants in healed and post-extraction sites [38]. A lower survival rate of only 80% after 11 years was found by Steyer et al. who used a comparable study setup [39]. In a clinical trial by Cannizzaro et al., no definite conclusion concerning the question if immediate loading would reduce implant failure [40]. However, they found high failure rates in immediately loaded implants when placed in post-extraction sites. Payer et al. investigated the bone level and soft tissue behavior of 20 implants made out of zirconium dioxide and found a success and survival rate of 95% after 24 months which is consistent with the results of the present study [41]. A significantly higher bone loss of 1 mm was found in the first year after implant placement, with the following bone loss being no longer significant after 24 months with an additional 0.3 mm. These observations are consistent with the results of the present study, with significantly higher marginal bone loss occurring in the first six months.

The majority of publications only treat implants in single-tooth gaps supported by single crowns [38–40]. In this study, 65 implants (47.1%) were supplied by splinted crowns whereas only 53 implants (38.4%) received single crowns. To the knowledge of the authors, this is the first study describing the outcome of multiple zirconia implants placed next to each other being restored by splinted crowns. Although this procedure showed good survival and success rates in this study, it should be verified by means of controlled prospective studies.

Immediately placed and loaded implants may provide better esthetic outcome and may be associated with lower bone loss and may also be favorable concerning soft tissue conditions [42, 43]. An ideal esthetic dental rehabilitation using implants is defined as the combination of a visual appealing restoration and healthy and harmonic contoured peri-implant soft tissue [44]. The Pink Esthetic Score developed by Fürhauser et al. has been recommended for evaluating peri-implant soft tissue and esthetics due to its reproducibility and easy and fast applicability [45]. In this study, a PES of 8.13 (baseline) was raised at the beginning, which improved to 10 after 24 months. The effect of a time-dependent rise in PES was also determined in a prospective study by Cosyn et al. [46]. They found an increase of PES from 10.67 in mean 3 months after implant placement to 11.67 after 6 months and 12.15 after 12 months following implant placement. A further study by Cosyn et al. considered the results of the Pink Esthetic Score to determine success of immediately placed and loaded titanium implants in the upper anterior jaw [47]. A result of ≤ 7 points was considered as a failure and a result of ≥ 12 points as a success. Five comparable studies with immediately loaded immediately placed implants resulted in a failure rate of 11% (PES ≤ 7)[46–49]. Fürhauser et al. found satisfactory esthetic long-term outcome in immediately placed and prosthetically restored implants [50]. The variables soft tissue texture and colour improved after one year. However, they noticed a mean mucosal recession of 0.26 ± 0.86 mm after 5 years. According to the criteria defined by Cosyn et al., only 2.3% of the implants assessed in this study are considered as failures. The PES results obtained in this study can be regarded as a success although prospective and controlled long-term studies are needed.

In the prospective part of this study, the oral health-related quality of life was recorded using a modified OHIP Edent questionnaire. In the present study, an average score of 0.54 points was obtained for the patient survey, suggesting high patient satisfaction (maximum score 100, low score = favorable satisfaction). Compared to other publications, this value suggests that ceramic implants may have comparable results to titanium implants regarding the improvement of quality of life after implant placement [51]. Other studies also concluded that immediate implant placement compared to conventional placement, as well as immediate compared to conventional loading, may yield similar results regarding patient satisfaction using the OHIP questionnaire [52, 53].

This study has several limitations. The recalls have been carried out irregularly and 47% of the patients only had a single recall. The third recall group (1–1.5 years post implant placement) contained only 14 patients with 45 implants. The lack of data in the 1–1.5-year recall group is a serious weakness of this study and limits the generalizability of the results in terms of long-term success. The results after the first year can be considered conclusive since the bone remodelling takes place primarily in the first year after implant placement and stabilizes thereafter [54]. Data about the healing process is lacking: for 7 out of 58 patients no data regarding bone and gingiva level could be acquired. Three patients did not show up for recall, four patients were not examined by radiographs and photography. These patients could not be included in the follow-up examination. However, it is possible that the outcome of the study could be affected both positively and negatively by including these patients. Another shortcoming is that 26.7% of the immediately loaded implants were not finally prosthetically rehabilitated at the end of the observation interval. The peri-implant bone topography was investigated using panoramic radiographs. This two-dimensional diagnostic procedure used for a three-dimensional structure is also a limitation because panoramic radiographs can only provide information about the mesial and distal peri-implant bone level. Especially the vestibular bone may show significant bone loss [55]. It must be taken into account that panoramic radiographs are not exactly reproducible, slight deviations in patient positioning can cause different views of the anatomical structures. This can cause measurement errors. However, panoramic radiographs have proven to be sufficient to examine the mesial and distal peri-implant bone level within an accuracy of 1 mm [56]. One treatment center used analog radiography; the data was digitized for analysis. The process of digitization may have a negative influence on the data accuracy. The Pink Esthetic Score of only 30 immediately placed implants in 13 patients was assessed, 45 implants could not be included due to lacking image quality, faulty camera settings or missing images, limiting the data significance. The retrospective character of this study is the reason why the survey of probing depth, sulcus fluid rate and implant mobility could not be standardized. However, according to Misch et al., the probing depth in particular is perceived as very subjective, which makes it a diagnostic criterion of comparatively low value [25].

## Conclusions

Taking into account the limitations of the study, zirconia dioxide implants were able to achieve similar results with respect to marginal radiological bone loss and patient satisfaction compared to titanium implants in this study regardless of the time point of placement and their type of loading. Especially in the esthetic zone, implants made of zirconium dioxide may be considered an alternative to conventional titanium implants due to their high esthetic potential. However, prospective controlled long-term studies are still needed.

## Supplementary Information

**Additional file1: Supplement 1:** Example for Pink Esthetic Score questionnaire

**Additional file2: Supplement 2:** OHIP questionnaire

## Data Availability

The data that was generated and analyzed for this study is not publicly available due to patient privacy. However, data will be available from the corresponding author upon reasonable request.
