# Supplementary material for: Success and patient satisfaction of immediately loaded zirconia implants with fixed restorations one year after loading
Source: BMC Oral Health. 2022 May 23;22:198. doi: 10.1186/s12903-022-02231-0 (PMC9125844; doi:10.1186/s12903-022-02231-0)
Supplement: Supplementary file 1 — Additional file1: Supplement 1: Example for Pink Esthetic Score questionnaire [file 12903_2022_2231_MOESM1_ESM.docx]

Example for Pink Esthetic Score questionnaire

Case 1:

Implant regio 43, picture above prior to treatment, picture below after implant placement and prosthetic rehabilitation


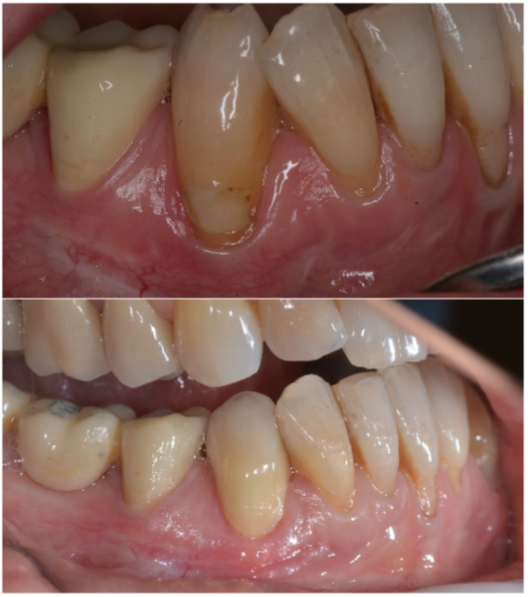


Score 0, 1 or 2

| Implant No. | Mesial papilla | Distal papilla | Height of marginal gingiva | Soft tissue contour | Alveolar processus deficiency | Soft tissue color | Soft tissue texture |
| --- | --- | --- | --- | --- | --- | --- | --- |
| 1 |  |  |  |  |  |  |  |
