# Supplementary material for: Success and patient satisfaction of immediately loaded zirconia implants with fixed restorations one year after loading
Source: BMC Oral Health. 2022 May 23;22:198. doi: 10.1186/s12903-022-02231-0 (PMC9125844; doi:10.1186/s12903-022-02231-0)
Supplement: Supplementary file 2 — Additional file2: Supplement 2: OHIP questionnaire [file 12903_2022_2231_MOESM2_ESM.docx]

OHIP questionnaire

Participant No.____________________

Please answer the following questions:

Do you smoke?

If yes, how many cigarettes per day? ___________ Since when? ___________

Do you grind your teeth?

Did you ever receive bisphosphonate medication?

If yes, when? ____________ For how long? _________________

Have you been diagnosed with osteoporosis?

Have you been diagnosed with diabetes?

Which medication have you been prescribed?

Please list name and dosage below:


Do you have any allergies or drug intolerances? If yes, list below:


Please tell us how content you are with your final implant supported prosthetic rehabilitation by answering the following questions:

| Since prosthetic rehabilitation: | Answers | | | | | | |
| --- | --- | --- | --- | --- | --- | --- | --- |
|  | always | very often | often | Some-times | seldom | | never |
| Did you have trouble chewing certain foods because of problems with your teeth, your dentures or other problems related to your oral cavity? | 5 | 4 | 3 | 2 | | 1 | 0 |
| Did you have problems with food debris stuck between your teeth or under your dentures? | 5 | 4 | 3 | 2 | | 1 | 0 |
| Did you feel like your dentures were not fitting properly? | 5 | 4 | 3 | 2 | | 1 | 0 |
| Have you been in pain in your oral cavity? | 5 | 4 | 3 | 2 | | 1 | 0 |
| Did you have trouble eating certain foods because of problems with your teeth, your dentures or other problems related to your oral cavity? | 5 | 4 | 3 | 2 | | 1 | 0 |
| Did you have sore spots in your oral cavity? | 5 | 4 | 3 | 2 | | 1 | 0 |
| Have you been worried because of because of problems with your teeth, your dentures or other problems related to your oral cavity? | 5 | 4 | 3 | 2 | | 1 | 0 |
| Have you felt insecure because of problems with your teeth, your dentures or other problems related to your oral cavity? | 5 | 4 | 3 | 2 | | 1 | 0 |
| Did you avoid certain foods because of problems with your teeth, your dentures or other problems related to your oral cavity? | 5 | 4 | 3 | 2 | | 1 | 0 |
| Have your eating habits been influenced in a negative way because of problems with your teeth, your dentures or other problems related to your oral cavity? | 5 | 4 | 3 | 2 | | 1 | 0 |
| Has it been impossible for you to eat with your dentures? | 5 | 4 | 3 | 2 | | 1 | 0 |
| Did you have to interrupt your meal because of problems with your teeth, your dentures or other problems related to your oral cavity? | 5 | 4 | 3 | 2 | | 1 | 0 |
| Have you been stressed because of problems with your teeth, your dentures or other problems related to your oral cavity? | 5 | 4 | 3 | 2 | | 1 | 0 |
| Have you ever been embarrassed because of problems with your teeth, your dentures or other problems related to your oral cavity? | 5 | 4 | 3 | 2 | | 1 | 0 |
| Have you avoided to socialize because of problems with your teeth, your dentures or other problems related to your oral cavity? | 5 | 4 | 3 | 2 | | 1 | 0 |
| Have you been less tolerant to your family or partner because of problems with your teeth, your dentures or other problems related to your oral cavity? | 5 | 4 | 3 | 2 | | 1 | 0 |
| Have you been short tempered when dealing with other persons because of problems with your teeth, your dentures or other problems related to your oral cavity? | 5 | 4 | 3 | 2 | | 1 | 0 |
| Have you been unable to enjoy others company because of problems with your teeth, your dentures or other problems related to your oral cavity? | 5 | 4 | 3 | 2 | | 1 | 0 |
| Did you have the feeling, your quality of life has been compromised because of problems with your teeth, your dentures or other problems related to your oral cavity? | 5 | 4 | 3 | 2 | | 1 | 0 |
